# Yeast-Derived Postbiotics as Emerging Candidates Against Enteric Bacterial Pathogens: Immunomodulatory and Antimicrobial Mechanisms Explored In Vitro

**DOI:** 10.3390/biology15161438

**Published:** 2026-08-20

**Authors:** Michelle Cerdán-Alduán, David García-Yoldi, Ana Ceniceros, Yadira Pastor, Raquel Conde-Álvarez

**Affiliations:** 1 Departments of Microbiology and Parasitology and of Molecular Biology, Navarra Medical Research Institute (IdiSNA), University of Navarra, 31008 Pamplona, Spain; mcerdan.3@alumni.unav.es; 2 LEV2050, Pol. Ind. Mutilva Baja, C. H, 31192 Mutilva Baja, Spain; dgarcia@lev2050.com (D.G.-Y.);

**Keywords:** yeast, postbiotics, heat-inactivated, immunomodulation, enteric infection, livestock

## Abstract

Among the many concerns surrounding global health, antimicrobial resistance (AMR) is widely recognized as a major threat, especially critical within livestock production, where restrictions on antibiotic use demand effective preventive alternatives. The documented health benefits and structural stability have positioned yeast-derived postbiotics as an attractive alternative, but research has largely focused on *Saccharomyces cerevisiae*, leaving non-*Saccharomyces* yeast species underexplored. To this end, in this study nine non-conventional yeast strains were selected and subjected to different thermal and chemical inactivation methods to determine the most suitable conditions for postbiotic obtention. Based on their physicochemical characterization and scalability potential, heat-treated postbiotics were selected for subsequent in vitro evaluation. Immunomodulatory assays demonstrated that heat-inactivated postbiotics from the different yeast strains were internalized by macrophages and induced dose-and-species-dependent expression of maturation markers CD40 and CD86, as well as TNF-α production, eliciting a proinflammatory response in vitro. Moreover, among all the species evaluated in this work, *Rhodotorula mucilaginosa* and *Wickerhamomyces anomalus* stood out for their ability to significantly reduce the adhesion of the enteropathogen enterotoxigenic *Escherichia coli* (ETEC) to intestinal cells in vitro. These results highlight the species-dependent immunomodulatory and anti-infective properties of selected yeast-derived postbiotics.

## Simple Summary

The livestock sector faces particular challenges due to the growing antimicrobial resistance, where restrictions on antibiotic use threaten both animal and human wellbeing. Owing to advantages such as enhanced safety and stability, yeast-derived postbiotics have increasingly been regarded as a compelling alternative to live microorganisms. In this study, we evaluate the postbiotic properties of nine yeast species, assessing their capacity to modulate the host immune response and interact with enteric pathogens. Our results demonstrate that selected yeast postbiotics are capable of eliciting a proinflammatory immune response and significantly reduce the adhesion of enterotoxigenic *Escherichia coli* to intestinal epithelial cells in vitro, highlighting the potential of yeast-derived postbiotics as preventive agents against bacterial enteric infections, representing a promising, antibiotic-free strategy with potential applications in animal health.

## 1. Introduction

Antimicrobial resistance (AMR) now poses a severe challenge to global health security, contributing substantially to mortality, morbidity, and imposing an immense burden of hospitalizations and economic losses worldwide [1]. The number of deaths associated with AMR was estimated of 5 million in 2019 [2], and has projected to increase to 10 million deaths and 130 million hospitalizations by 2050 [3]. AMR is primarily driven by excessive and inappropriate applications of antibiotics in human medicine, veterinary practice, and agricultural settings, fostering the selection and proliferation of multidrug-resistant organisms [3,4]. In parallel, the release of antibiotic residues into the environment contaminates soils, waterways, and wildlife animals, promoting the horizontal transfer of resistance genes [4]. As a result, AMR has evolved into a multifaceted One Health crisis that compromises clinical, veterinary, and ecological ecosystems [4,5].

Within this framework, one of the core strategies to combat AMR is the implementation of robust infection prevention and control programs, particularly in economically developing nations with restricted healthcare capacities [2,6]. Preventive measures are of particular importance in the livestock sector, as limitations on antibiotic use compromise animal welfare and food security, and, by extension, human health [7]. Beyond conventional preventive measures such as vaccination and sanitation, complementary approaches aimed at strengthening host resistance are gaining attention as critical strategies to safeguard vulnerable patients and reduce healthcare burdens [2,6,8].

Among these approaches, administering probiotics represents an increasingly viable pathway toward promoting overall host health and have been widely employed in animal health due to their well-documented beneficial effects [9,10]. Nevertheless, as they consist of living organisms, their use carries certain risks and drawbacks: horizontal transfer of resistance genes to other microorganisms, opportunistic infections in immunocompromised or stressed animals, poor survival and colonization and stability loss during long-term storage [11,12,13].

To overcome these limitations, attention has recently been focused on postbiotics, which offer a safer profile and superior stability. Postbiotics are defined as preparations of inactivated microorganisms and/or their metabolites that confer health benefits to the host [14]. Their beneficial effects have been largely described in the literature attributing them antimicrobial properties, antioxidant properties or immunomodulatory properties [14,15].

To date, bacterial species remain the most studied microorganisms and primary source of postbiotics, including *Lactobacillus*, *Bifidobacterium* and *Diploccoccus* [16]. However, increasing evidence highlights yeasts as valuable and complementary alternative sources of postbiotics, since they exhibit greater resistance to gastrointestinal enzymes, bile salts, and variations in pH and temperature [16]. While *Saccharomyces cerevisiae* remains the most studied yeast, non-conventional species have attracted growing interest due to their diverse biotechnological potential and functional properties [17]. Recent studies with both *Saccharomyces* and non-*Saccharomyces* postbiotics demonstrated that yeast postbiotics beneficially modulate host immunity, pathogen interaction, gut barrier integrity, and microbiota composition [16]. Moreover, the application of yeast-derived postbiotics in livestock has been a topic of rising relevance, showing promising results in animal health [16].

The immunomodulatory potential of yeast-derived postbiotics is primarily attributed to cell wall components (mannoproteins, β-glucans, and chitin) which interact with the host immune system [16,18], triggering trained innate immunity [16,19]. These components demonstrate their capacity to elicit a proinflammatory response by enhancing the expression of cell maturation surface markers such as CD40 and CD86 [20,21,22,23], and increasing the production of cytokines, such as TNF-α [16,23,24]. Importantly, several studies have also shown the direct interaction of yeast-derived postbiotics with enteric pathogens, blocking the adhesion to host cells and thereby avoiding bacterial colonization [16,18,25].

This study aims to develop yeast-derived postbiotics by evaluating and optimizing different inactivation strategies for nine different yeast species to identify an effective and scalable manufacturing approach. Furthermore, the resulting postbiotics are selected for subsequent in vitro evaluation to assess their capacity to activate immune cells and reduce the adhesion of enterotoxigenic *Escherichia coli* (ETEC) to intestinal epithelial cells. Through this approach, this research seeks to establish a foundation for utilizing yeast-derived postbiotics as functional ingredients to prevent enteric bacterial diseases.

## 2. Materials and Methods

### 2.1. Yeast Strains, Growth Conditions and Inactivation Methods

In this study, nine yeast species were selected based on previous studies from the group [18], as well as their beneficial properties described in the literature, as summarized in Table 1. The strains, originally isolated from agri-food sources, were provided by LEV2050 (https://www.lev2050.com/, Pamplona, Spain) and are deposited in their cellbank, where they are not publicly available. These strains were stored in Cryobeads (Microkit laboratories, Madrid, Spain) at −80 °C until use. Yeasts were grown in YPD medium containing 1% yeast extract (Condalab, Madrid, Spain), 2% peptone (Condalab, Madrid, Spain), and 2% dextrose (Panreac-BioChemica, Barcelona, Spain) for 48 h under constant agitation conditions (180 rpm) at 28 °C. YPD agar plates containing 1.5% agar were also used where indicated.

To establish the optimum inactivation conditions for large-scale and real-product manufacturing, yeast cells were inactivated employing either thermal treatment (HT) at temperatures ranging from 60 to 80 °C for varying durations, or chemical inactivation using 2-bromoethylamine hydrobromide and formaldehyde (BEI/FA) as previously described [18,58]. To improve the thermal inactivation conditions previously reported by our group [18], a screening phase was conducted to identify the lowest possible temperature capable of achieving complete inactivation, given the relevance of milder thermal conditions for preserving cell wall integrity and facilitating scale-up. For this purpose, temperatures ranging from 50 to 100 °C combined with exposure times ranging from 20 to 80 min were tested for each yeast species. Complete yeast inactivation, defined as the absence of visible colony growth, was verified by plating of treated cultures on YPD agar plates and incubation at 28 °C for 5 days. Inactivated cells were then centrifuged at 6000× *g* for 20 min, washed with ultrapure water, lyophilized, and stored for further use.

### 2.2. Characterization

#### 2.2.1. Size and Zeta Potential Determination

To evaluate these changes and compare the effects of the two inactivation methods, each lyophilized yeast strain was resuspended in ultrapure water at a concentration of 1 mg/mL and analyzed using a Mastersizer (Malvern Instruments, Malvern, UK) to determine particle size, and a Zetasizer (Brookhaven Instruments Corporation, Holtsville, NY, USA) to measure zeta potential. 

#### 2.2.2. Presence of β—Glucans, Mannoproteins and Chitin

Inactivated yeast samples were analyzed for 1,3–1,6 β-glucan content using the Megazyme yeast and mushroom kit (K-YBGL) (Megazyme Ltd., Bray, Co. Wicklow, Ireland) following the manufacturer’s instructions. Briefly, β-glucans were solubilized in 12 M H_2_SO_4_ for 1 h at 30 °C, and subsequently, hydrolyzed into glucose at 100 °C for another 2 h. Residual glucose fragments were oxidized using a combination of glucose oxidase and peroxidase, enabling quantification of total β-glucan content. To determine the α-glucan content, samples were enzymatically hydrolyzed. Glucose concentration was measured using a glucose oxidase peroxidase GOPOD reagent. The β-glucan content was calculated as the difference between total glucan and α-glucan.

The presence of mannoproteins and chitin was evaluated following the protocol described by Cerdán-Alduán et al. [18]. Briefly, inactivated yeast cells were stained using Concanavalin A (ConA) conjugated with Alexa Fluor 488 and wheat-germ agglutinin (WGA) conjugated with Alexa Fluor 488, which bind specifically to mannoproteins and chitin, respectively. Following the staining protocol yeast cells were analyzed by Flow cytometry (Attune Cytometer, Applied Biosystem, Waltham, MA, USA) to assess mean fluorescence intensity (MFI).

### 2.3. Cellular Studies

#### 2.3.1. Cell Lines and Growth Conditions

For this study, three different cell lines were employed: HeLa, RAW 264.7 and Caco-2 cells. HeLa and RAW 264.7 were incubated in RPMI-1640 medium (Gibco^®^, Waltham, MA, USA), while Caco-2 cells were maintained in DMEM medium (Gibco^®^, Waltham, MA, USA). Both media were previously supplemented with 10% FBS (Gibco^®^, Waltham, MA, USA) and 1% penicillin/streptomycin (Gibco^®^, Waltham, MA, USA). All cell lines were maintained at 37 °C in a humidified atmosphere containing 5% CO_2_.

#### 2.3.2. Cytotoxicity in HeLa and RAW 264.7 Cells

The potential cell toxicity exerted by thermal-treated yeast postbiotics was evaluated using the MTT method in both HeLa cells and RAW 264.7 macrophages. Briefly, RAW 264.7 macrophages or HeLa cells (10^4^ cells/well) were incubated in 96-well flat bottom microplates (Falcon) for 24 h in complete RPMI medium. Since whole heat-inactivated yeast cells were used in this assay, yeast concentrations were standardized in colony-forming units per milliliter (CFU/mL), consistent with previous studies [59]. Cells were then exposed to serial 1:2 dilutions of heat-inactivated yeast strains ranging from 1.25 × 10^7^ to 7.8 × 10^5^ CFU/mL (corresponding to cell-postbiotic ratios of 1:125–1:7.8) in RPMI medium and incubated for 24 h. A 10% MTT solution in RPMI medium was added to wells and following 4 h of incubation, supernatants were removed, and pure DMSO (Panreac, Barcelona, Spain) was added to samples. Absorbance was measured at 540 nm (Thermo Scientific, Waltham, MA, USA).

#### 2.3.3. Yeast-Derived Postbiotics Uptake by RAW 264.7 Cells

The cellular uptake of rhodamine-stained yeast postbiotics by RAW 264.7 macrophages was assessed by fluorescence microscopy and flow cytometry.

For this purpose, a non-confluent monolayer of RAW 264.7 cells was seeded at 1 × 10^5^ cells/well in 24-well flat microplates (Corning^®^ Costar^®^ TC-treated, Corning, NY, USA) and incubated for 24 h in RPMI medium. For fluorescence microscopy, glass coverslips were sequentially treated with acetone, absolute ethanol and then autoclaved. Sterilized coverslips were placed in each well prior to cell seeding to facilitate subsequent imaging of the cell monolayer.

Inactivated yeast cells (2 × 10^6^ CFU/mL) were stained with 1 µL of a 100 µg/mL rhodamine solution for 30 min at 600 rpm in the dark. Subsequently, the cells were resuspended in PBS and centrifuged to eliminate the excess dye. The rhodamine labeled yeasts were added to each well containing RAW 264.7 cells and incubated for additional 24 h.

For flow cytometry analysis, cells were detached using 500 µL of a PBS containing 2% of bovine serum albumin (BSA). A viability stain was employed in all the samples before analyzing by flow cytometry (Attune Cytometer, Applied Biosystem, Waltham, MA, USA).

For fluorescence microscopy, the culture media was removed from the well and washed 3 times with PBS. Cells attached to the coverslip were then fixed with 100 µL of 4% paraformaldehyde for 30 min. After fixation, wells were washed 3 times with PBS, and coverslips were gently removed and mounted on microscope slides containing a 10 µL drop of DAPI I counterstain (30-804930, Abbott Molecular, Des Plaines, IL, USA) to stain macrophage nuclei. Samples were examined using a Zeiss Axiolab 5 microscope (Zeiss, Oberkochen, Germany), and images were acquired and processed with an Axiocam 202 camera and ZEN 3.0 (blue edition) software (Zeiss, Oberkochen, Germany).

#### 2.3.4. Expression of Surface Markers in RAW 264.7

The capacity of heat-inactivated yeast-derived postbiotics to modulate the expression proinflammatory cell surface markers CD40 and CD86 was investigated on RAW 264.7 macrophages.

Accordingly, RAW 264. 7 cells were seeded at 1 × 10^5^ cells/well in 24-well plates (Corning^®^ Costar^®^ TC-treated, Corning, NY, USA) and incubated for 24 h in RPMI medium. Following incubation, heat-inactivated yeast postbiotics were adjusted to final concentrations of 2 × 10^6^ and 4 × 10^6^ CFU/mL (1:10 and 1:20 macrophage–postbiotic ratio per well) and added to the wells for 24 h. Cells were detached employing PBS containing 2% BSA and incubated with CD16/32 (2.5 µg/mL) blocking antibody (Biosciences, Cat. No. 553142) solution for 15 min in the dark at 4 °C. For surface staining a solution that contained the following anti-mouse, fluorochrome labeled antibodies was added to the cells: CD40 (5 µg/mL) (Biolegend, Cat. No. 124608), CD86 (2 µg/mL) (Biolegend, Cat. No. 105016) and a live/dead stainer (2.5 µM) (Biolegend, Cat. No. 425301) for 15 min in the dark at 4 °C. The samples were analyzed by Flow Cytometry (Attune Cytometer, Applied Biosystem, Waltham, MA, USA).

#### 2.3.5. Cytokine Production in RAW 264.7

RAW 264.7 macrophages (1 × 10^5^ cells/well) were incubated with heat treated yeast postbiotics at 2 × 10^6^ and 4 × 10^6^ CFU/mL (corresponding to 1:10 and 1:20 macrophage–postbiotic ratio per well) for 24 h in 24-well plates (Corning^®^ Costar^®^ TC-treated, Corning, NY, USA). Subsequently, cell culture supernatants were collected to determine TNF-α concentrations by enzyme-linked immunosorbent assay (ELISA) using the Mouse TNF-α DuoSet kit (#DY410, R&D Systems, Minneapolis, MN, USA), following manufacturer’s instructions. Briefly, 96-well flat-bottom MaxiSorp™ plates (Cat. No. 442404, Thermo Fisher Scientific, Waltham, MA, USA) were coated with the capture antibody overnight at room temperature, blocked for 1 h, and incubated with samples and standards for 2 h. Detection antibody was then added for 2 h, followed by streptavidin-HRP incubation for 20 min. The colorimetric reaction was developed using substrate solution, and absorbance was measured at 405 nm.

#### 2.3.6. Adhesion Assay in Caco-2 Cells

Bacterial cell adhesion prevention through yeast postbiotic coaggregation was tested based on the methodology established by Cerdán-Alduán et al. [18] with some modifications. Caco-2 cells (2 × 10^5^ cells/well) were planted into 24-well plates (Corning^®^ Costar^®^ TC-treated, Corning, NY, USA) and incubated for 72 h in DMEM medium to obtain a non-confluent monolayer. Following the protocol, Enterotoxigenic *Escherichia coli* H10407 (ATCC 35401) was employed for this assay. Pre-cultures of ETEC were grown for 12 h to the stationary phase in tryptic soy broth (TSB) medium at 37 °C and diluted to reach 2 × 10^6^ CFU/mL. For this assay, heat-inactivated yeast postbiotics from *M. pulcherrima*, *P. fermentans*, *R. mucilaginosa*, *S. cerevisiae*, and *W. anomalus* were selected for this assay based on their previously demonstrated capacity to agglutinate with ETEC [18]. The yeast postbiotics were adjusted to a concentration of 2 × 10^5^ CFU/mL. A ratio of 10:1 of bacteria–yeast mixture was prepared in an Eppendorf tube and incubated for 30 min at room temperature. Caco-2 cells were then washed twice with PBS, and a volume of 450 μL of the mixture was added to each well, followed by centrifugation at 900× *g* for 10 min to facilitate the encounters of microorganisms. The plates were then incubated at 37 °C with 5% CO_2_ for 2 h. After incubation, Caco-2 cells were washed with PBS and lysed using a 1% Triton X-100 solution (Sigma, Madrid, Spain) at room temperature. The resulting lysates were serially diluted and plated on TSA for viable bacterial colony counting.

### 2.4. Statistical Analysis

Statistical analyses were performed using the computer software GraphPad Prism v.9.2.0.332 (GraphPad, San Diego, CA, USA). The significant differences were determined by one-way or two-way ANOVA, followed by Dunnett’s post hoc test, as appropriate.

## 3. Results and Discussion

### 3.1. Yeast Inactivation

**Figure A1:**
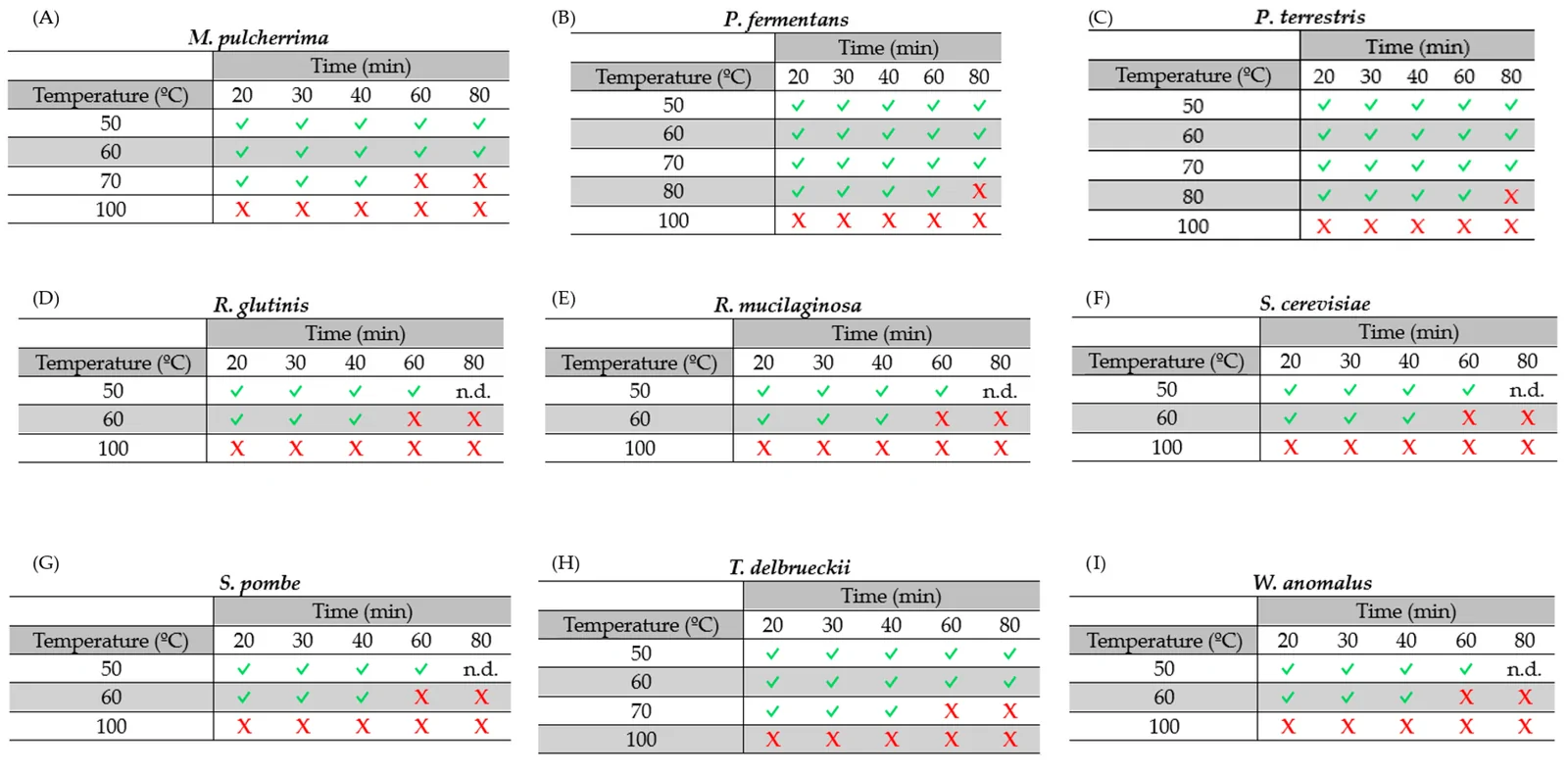
Thermal inactivation screening results. A screening process was made to refine the thermal inactivation conditions in order to obtain the lowest possible temperature capable of achieving complete inactivation for each yeast species (**A**) *M. pulcherrima*, (**B**) *P. fermentans*, (**C**) *P. terrestris*, (**D**) *R. glutinis*, (**E**) *R. mucilaginosa*, (**F**) *S. cerevisiae*, (**G**) *S. pombe*, (**H**) *T. delbrueckii* and (**I**) *W. anomalus*. Tested temperatures ranged from 50 to 100 °C in combination with exposure times ranging from 20 to 80 min. Complete inactivation was defined as the absence of visible colony growth after plating on YPD agar and incubation at 28 °C for 5 days, indicated by (X). The presence of colonies, indicating incomplete inactivation, is indicated by (✓). Conditions that were not tested for a given species are indicated as not determined (n.d.).

**Figure A2:**
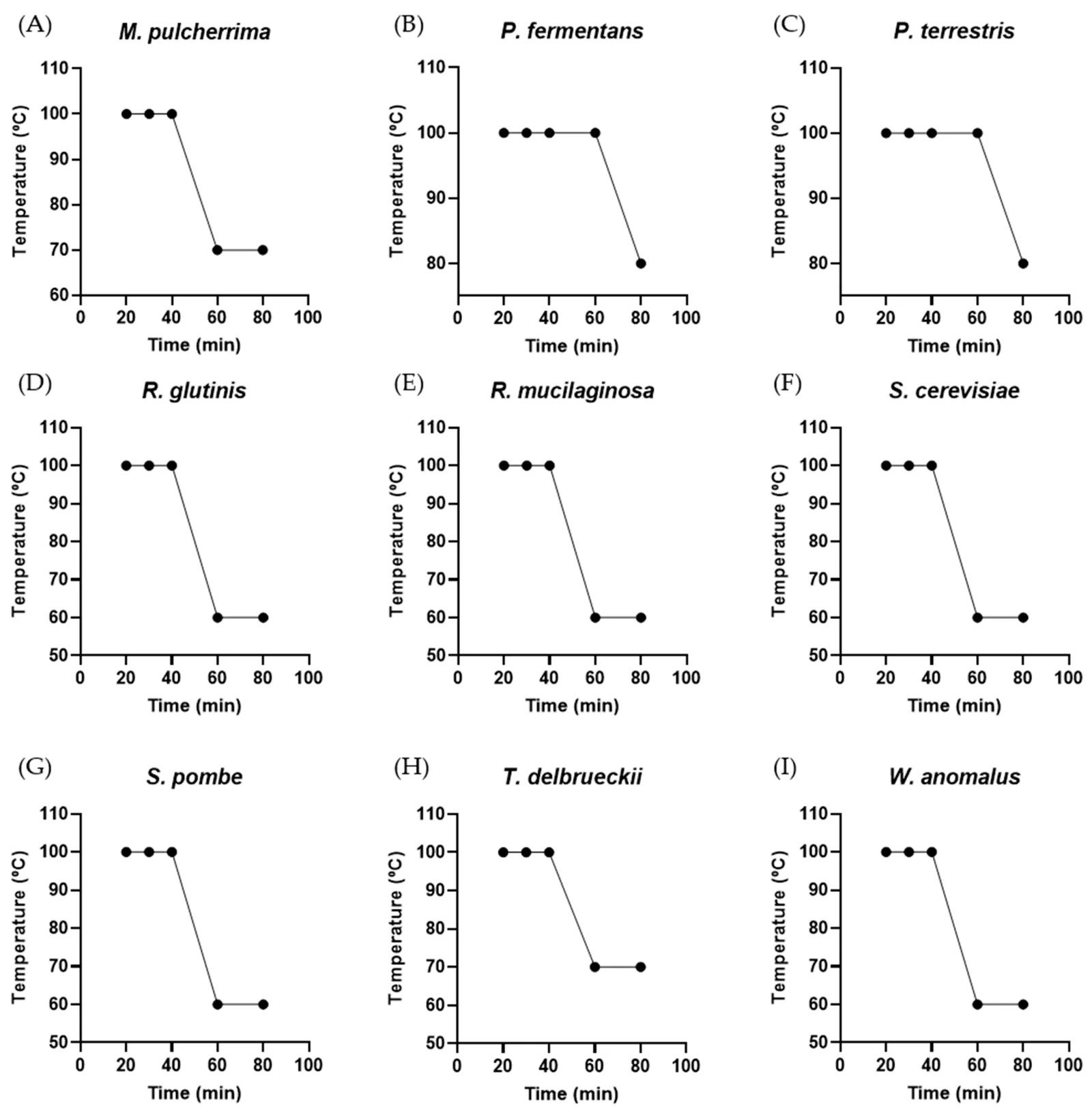
Representation of the thermal inactivation screening results. Graphs show the thermal inactivation profiles for each yeast species evaluated in this study (**A**) *M. pulcherrima*, (**B**) *P. fermentans*, (**C**) *P. terrestris*, (**D**) *R. glutinis*, (**E**) *R. mucilaginosa*, (**F**) *S. cerevisiae*, (**G**) *S. pombe*, (**H**) *T. delbrueckii* and (**I**) *W. anomalus*. For each tested exposure time the minimum temperature required to achieve complete inactivation is shown, defined as the absence of visible colony growth.

Building upon the protocol described by Cerdán-Alduán et al. (2025) [18] two inactivation methods were evaluated against stationary-phase yeast cultures: one based on heat treatment and another based on a chemical treatment. A key feature of these protocols is the use of whole inactivated cells, rather than cell extracts or purified fractions [18]. In the present work, with a view toward industrial scale-up and preservation of yeast cell wall components, the primary objective was to refine the minimum inactivation temperatures previously reported by our group, prioritizing lower thermal exposure over shorter processing times, in order to ensure greater process reproducibility, efficiency, and feasibility under large-scale production settings. Heat treatment conditions were refined for *Metschnikowia pulcherrima*, *Pichia fermentans*, *Papiliotrema terrestris* and *Torulaspora delbrueckii* (Table 2), while the inactivation conditions for the remaining yeast species were retained as described in the original protocol [18]. The full screening dataset supporting this selection is provided in Appendix A section (Figure A1 and Figure A2).

### 3.2. Characterization of the Yeast Inactivated Postbiotics

**Figure 1:**
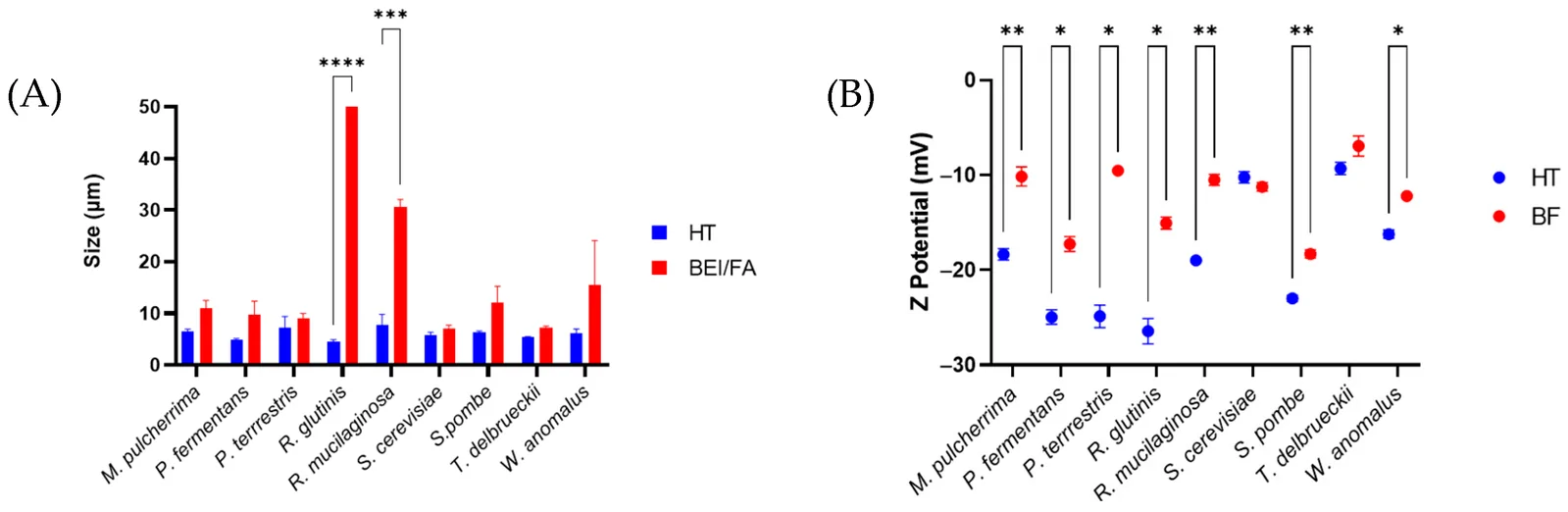
Physicochemical characterization of yeast-derived postbiotics obtained by heat treatment (HT) and chemical inactivation (BF). (**A**) Particle size distribution (µm) and (**B**) zeta potential (mV) of postbiotic preparations from the nine yeast species (* *p* < 0.05, ** *p* < 0.01, *** *p* < 0.001, **** *p* < 0.0001). Error bars represent SEM (*n* = 3).

One of the potential side effects of yeast inactivation is the alteration of cell size and net surface charge, which may, in turn, influence downstream immunomodulatory interactions. To assess the structural changes induced by each treatment and to enable direct comparison, cell size was determined in inactivated yeast postbiotics using a mastersizer particle size analyzer. Results demonstrated that the postbiotic material obtained by heat treatment (HT) exhibited a smaller particle size than that obtained by chemical treatment (Figure 1A). In this sense, Almeida et al. demonstrated that reduced particle size increased phagocytosis in a *Paracoccidioides brasiliensis* mutant, thereby promoting an immune response [60], suggesting that the size differences observed may have important immunological implications. Notably, thermal stress has been shown to induce significant structural reorganization in *S. cerevisiae*, including changes in organelle size and morphology [61]. Although these observations were made under mild, sub-lethal conditions (38 °C), it is plausible that the heat treatment applied here also produced an irreversible structural collapse, potentially accounting for the reduced particle size observed in our heat-inactivated preparations. Regarding the particularly pronounced size increases observed in chemically inactivated (BF) particles of *R. glutinis* and *R. mucilaginosa*, an additional explanation may be related to flocculation, a process by which yeast cells aggregate through lectin-like interactions mediated by Flo1-family proteins [62].

We also determined the net surface charge of the particles by measuring their zeta potential, providing insight into their colloidal stability and potential interactions with biological environments. As shown in Figure 1B, HT postbiotic material consistently exhibited more negative zeta potential values compared to BF counterparts in six of the nine yeast species evaluated: *M. pulcherrima*, *P. fermentans*, *P. terrestris*, *R. glutinis*, *R. mucilaginosa*, *S. pombe*, and *W. anomalus*. In contrast, no statistically significant differences between inactivation methods were observed for *S. cerevisiae* and *T. delbrueckii*. Although demonstrated in the context of diesel exhaust particles, evidence that zeta potential is a primary determinant of bioreactivity in human immune cells [63] suggests that the more negative surface charge of heat-inactivated postbiotics may similarly promote immune cell interaction and contribute to their immunomodulatory potential.

As mentioned above, the immunomodulatory potential of inactivated yeasts relies largely on cell wall components such as β-glucans, mannoproteins, and chitin, which are recognized by pattern recognition receptors (PRRs) on immune cells as microbe-associated molecular patterns (MAMPs), triggering both innate immune responses and trained innate immunity [16,18]. Given that inactivation treatments may alter the structural integrity and composition of the cell wall, potentially affecting these bioactive properties, changes in cell wall composition induced by both heat and chemical treatments were assessed in the yeast-derived postbiotics obtained. β-glucans were determined using an enzymatic colorimetric kit, while mannoproteins and chitin were assessed by flow cytometry following fluorescent staining of the yeast-derived postbiotics to evaluate mean fluorescence intensity (MFI) (see Table A1). The presence of the three main yeast cell wall components (β-glucans, mannoproteins, and chitin) was confirmed in all postbiotic preparations, regardless of the inactivation method applied.

Based on the retention of key structural components and its suitability for large-scale production, heat-inactivated yeast preparations were selected for subsequent assays. Unlike chemical methods, heat inactivation does not require additional reagents or downstream removal steps, facilitating industrial implementation and improved cost-effectiveness while maintaining key yeast cell integrity [64,65].

### 3.3. MTT Cytotoxicity Assay in HeLa and RAW 264.7 Cells

**Figure 2:**
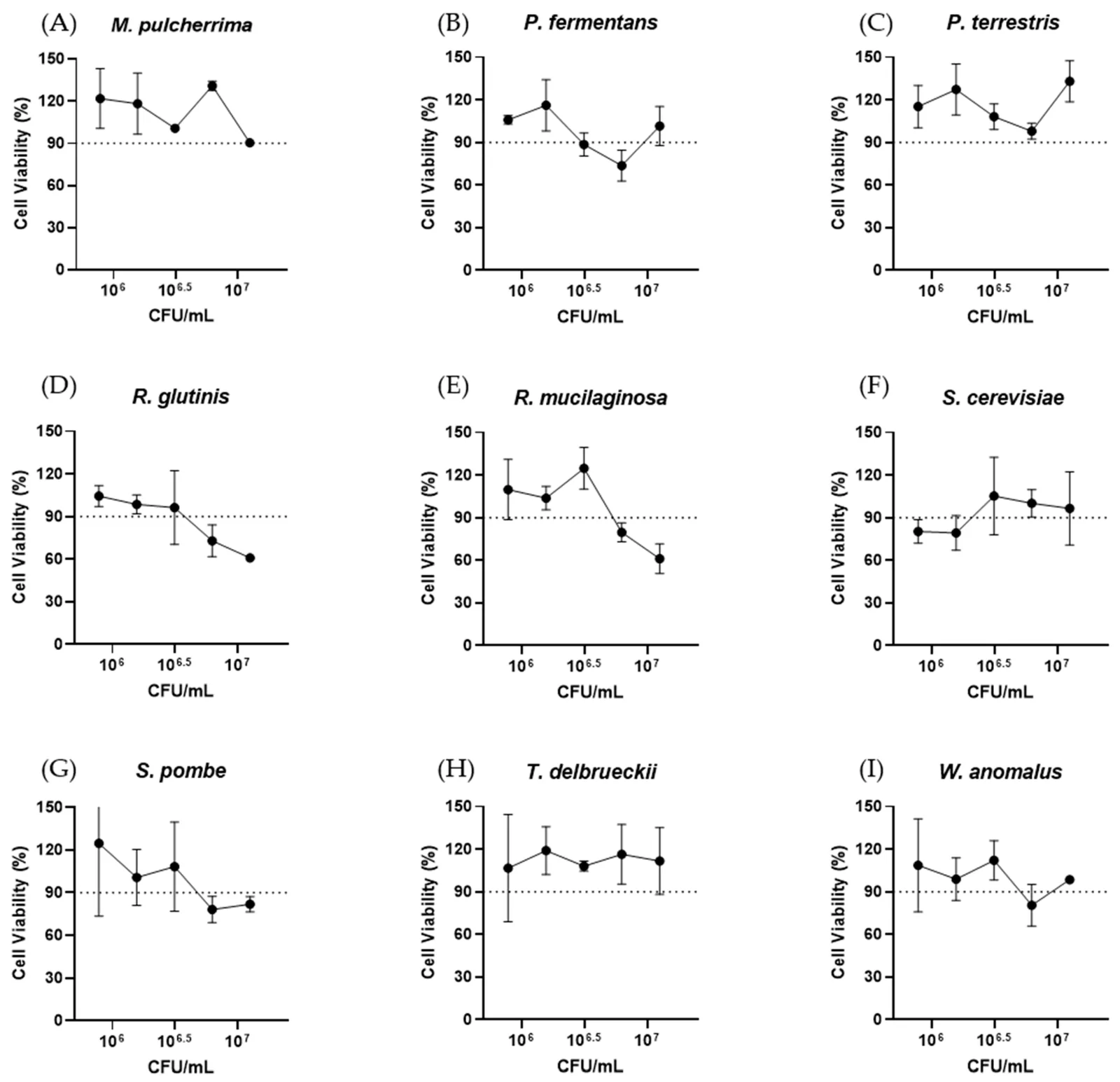
Effect of heat-inactivated yeast postbiotics on HeLa cell viability. Cell viability (%) was assessed by MTT assay in HeLa cells after 24 h of incubation with increasing concentrations of heat-inactivated expressed as CFU/mL (**A**) *M. pulcherrima*, (**B**) *P. fermentans*, (**C**) *P. terrestris*, (**D**) *R. glutinis*, (**E**) *R. mucilaginosa*, (**F**) *S. cerevisiae*, (**G**) *S. pombe*, (**H**) *T. delbrueckii* and (**I**) *W. anomalus*. The dashed line indicates the 90% cell viability threshold used as a reference. Experiment was performed in triplicate. Error bars represent SEM.

In order to evaluate the cytotoxicity of heat-inactivated yeast postbiotics, HeLa and RAW 264.7 cells and were treated with different concentrations of the compounds (material obtained from c.a 7.8 × 10^5^ to 1.25 × 10^7^ CFU/mL) and incubated for 24 h. First, we evaluated the cytotoxicity of our yeast-derived postbiotics in HeLa cells as an epithelial model representing the first barrier encountered by the postbiotics. Overall, most postbiotics maintained HeLa cell viability above 90% across all concentration ranges tested. These findings align with the recognized probiotic properties of these strains [17,27,35,39,41,44,52,66,67]; indeed, *S. cerevisiae*, *S. pombe*, and *W. anomalus* hold Qualified Presumption of Safety (QPS) status, corroborating their established safety [48]. However, the response was species-dependent, showing a dose-dependent trend in viability in some cases (Figure 2), as is the case for *Rhodotorula* species. Both *R. glutinis* and *R. mucilaginosa* showed reduced cell viability at the highest concentrations tested (Figure 2). Likewise, variability in cell viability was observed for *P. fermentans* and *P. terrestris*. This phenomenon may be partly attributed to the production of carotenoids by *Rhodotorula* species and exopolysaccharides by *P. fermentans* and *P. terrestris* [40,68,69,70,71]. Finally, the dose-dependent effect observed in *M. pulcherrima* might be associated with the production of pulcherrimin, an iron-chelating pigment with well-documented antimicrobial properties and cytotoxic effects in different cell lines [72,73].

**Figure A3:**
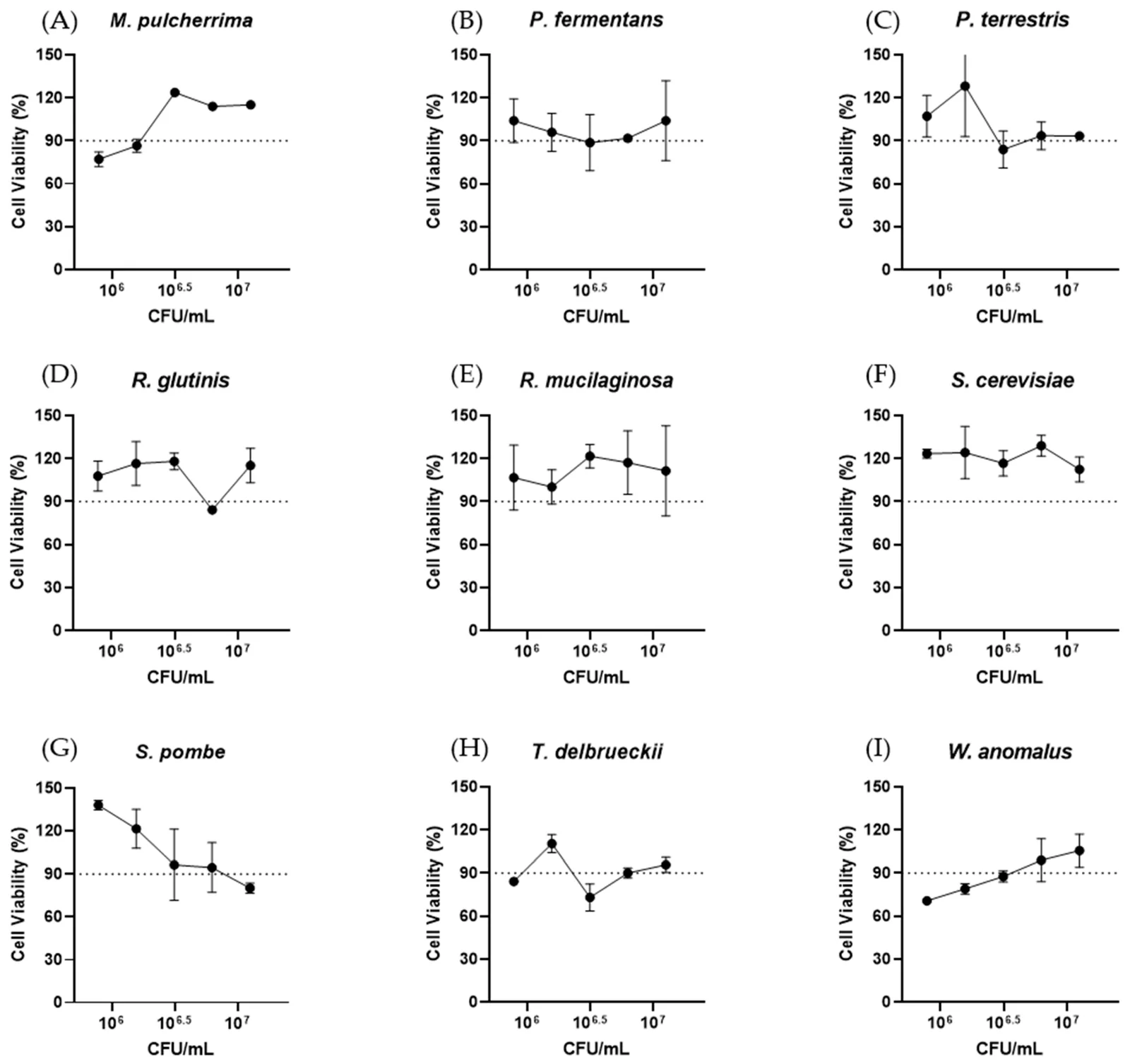
Effect of heat-inactivated yeast postbiotics on RAW 264.7 macrophage cell viability. Cell viability (%) was assessed by MTT assay in RAW 264.7 cells after 24 h of incubation with increasing concentrations of heat-inactivated expressed as CFU/mL (**A**) *M. pulcherrima*, (**B**) *P. fermentans*, (**C**) *P. terrestris,* (**D**) *R. glutinis*, (**E**) *R. mucilaginosa*, (**F**) *S. cerevisiae*, (**G**) *S. pombe*, (**H**) *T. delbrueckii* and (**I**) *W. anomalus*. Experiment was performed in triplicate. Error bars represent SEM (when not visible, they are within the symbol).

Moreover, we tested cell viability in RAW 264.7 macrophages to confirm that the postbiotics neither compromised cell viability in this model nor confounded the assessment of their immunomodulatory effects. Consistent with the results obtained in HeLa, cell viability in RAW 264.7 macrophages remained above 90% at most concentrations tested across all postbiotics (Figure A3). These findings are in line with the current literature which describes how bioactive compounds, such as β-glucans, exopolysaccharides and zymosan, derived from *S. cerevisiae*, maintain cell viability above 90% at concentrations of 5–20 µg/mL, 100–200 ug/mL, and 20–100 µg/mL, respectively [74,75,76].

All in all, this assay demonstrated that the preparations exhibit no cytotoxicity at dilutions equivalent to concentrations below 3.13 × 10^6^ CFU/mL and consequently, these non-toxic levels were selected for subsequent experiments (i.e., to cell-to-postbiotic ratios of 1:20 and 1:10).

### 3.4. Yeast-Derived Postbiotics Uptake

Given the well-characterized interaction between yeast cell wall components and PRRs (such as Dectin 1, complement receptor 3, mannose receptor or TLR2) expressed on macrophages [16], we sought to investigate whether these interactions were sufficient to drive the phagocytosis of heat-inactivated yeast postbiotics by macrophages, despite their relatively large size compared to other microorganisms. To evaluate this, RAW 264.7 cells were incubated with rhodamine-labeled, heat-inactivated yeast postbiotics. Macrophages were subsequently analyzed by flow cytometry to assess both the proportion of cells positive for postbiotic uptake and the relative number of internalized particles per macrophage.

**Figure 3:**
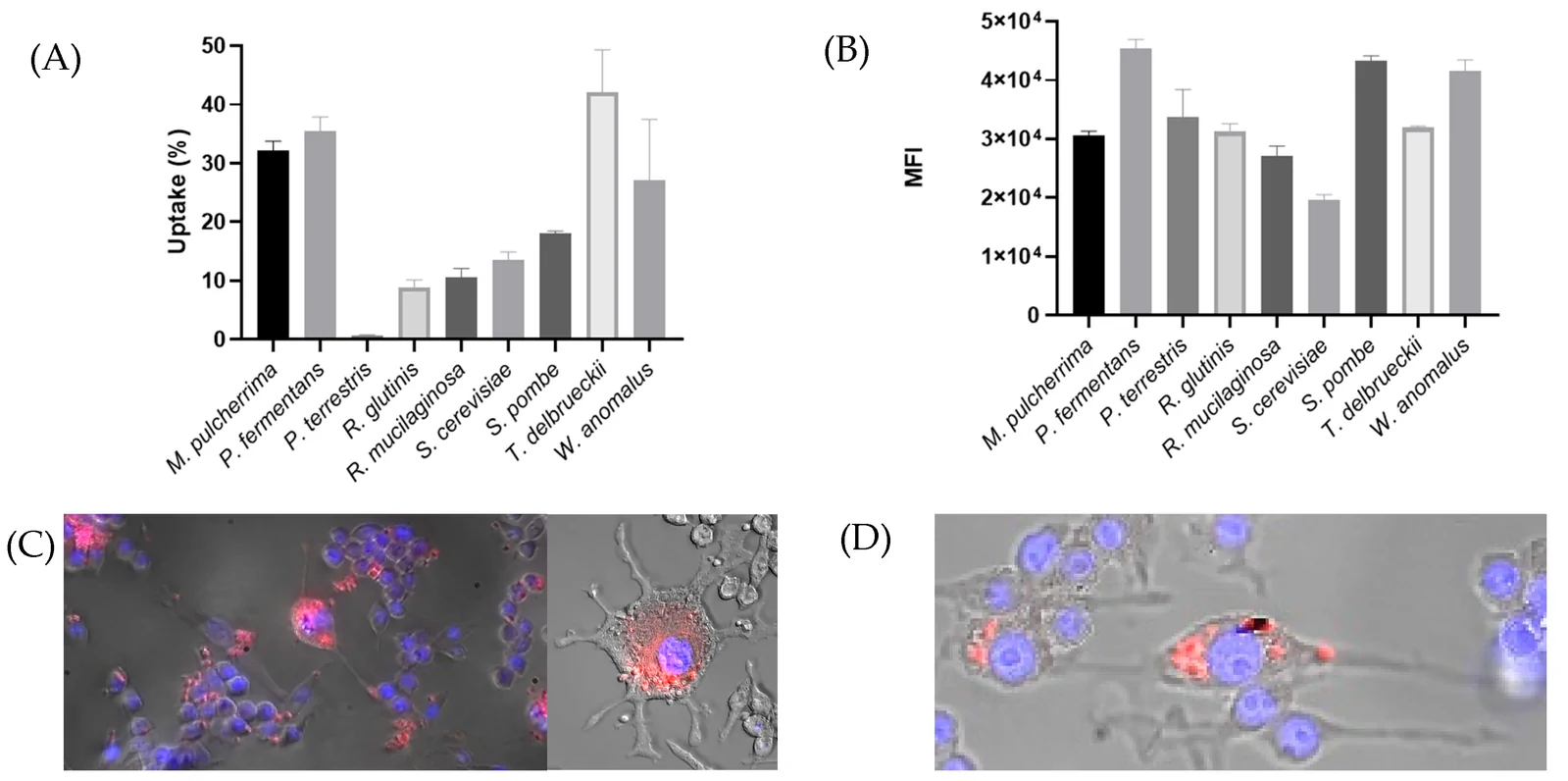
Flow cytometry and fluorescence microscopy analysis of postbiotic internalization by RAW 264.7 macrophages. (**A**) Percentage (%) of macrophages positive for postbiotic uptake and (**B**) mean fluorescence intensity (MFI) per cell, reflecting the relative number of postbiotic particles internalized per macrophage. Error bars represent SD (*n* = 3). Representative fluorescence images illustrating macrophage internalization of heat-treated (HT) postbiotics derived from (**C**) *R. mucilaginosa* and (**D**) *W. anomalus*.

Heat-inactivated postbiotics from all yeast species tested were captured by macrophages (Figure 3A), with the percentage of macrophages positive for internalization ranging from 9% to 42% across the different species. *P. fermentans*, *T. delbrueckii*, and *W. anomalus* showed the highest percentages of uptake, whereas *P. terrestris* exhibited minimal uptake levels. Furthermore, mean fluorescence intensity (MFI) analysis revealed that macrophages were capable of internalizing multiple postbiotic particles per cell across all species examined (Figure 3B). MFI values ranged from 2 × 10^4^ to 4.5 × 10^4^, with the highest values observed for *P. fermentans*, *S. pombe*, and *W. anomalus*, and the lowest for *S. cerevisiae*. These findings were corroborated by fluorescence microscopy; representative images of RAW 264.7 macrophages with internalized heat-treated *R. mucilaginosa* and *W. anomalus* postbiotics are shown in Figure 3C and 3D, respectively. These results are consistent with the available literature, which has previously described the uptake of various isolated bioactive cell wall components or live yeast cells by macrophages, from either *S. cerevisiae* or *Candida albicans*, thereby initiating downstream activation of the innate immune response [77,78,79,80,81,82,83].

### 3.5. In Vitro Immune Cell Activation

**Figure 4:**
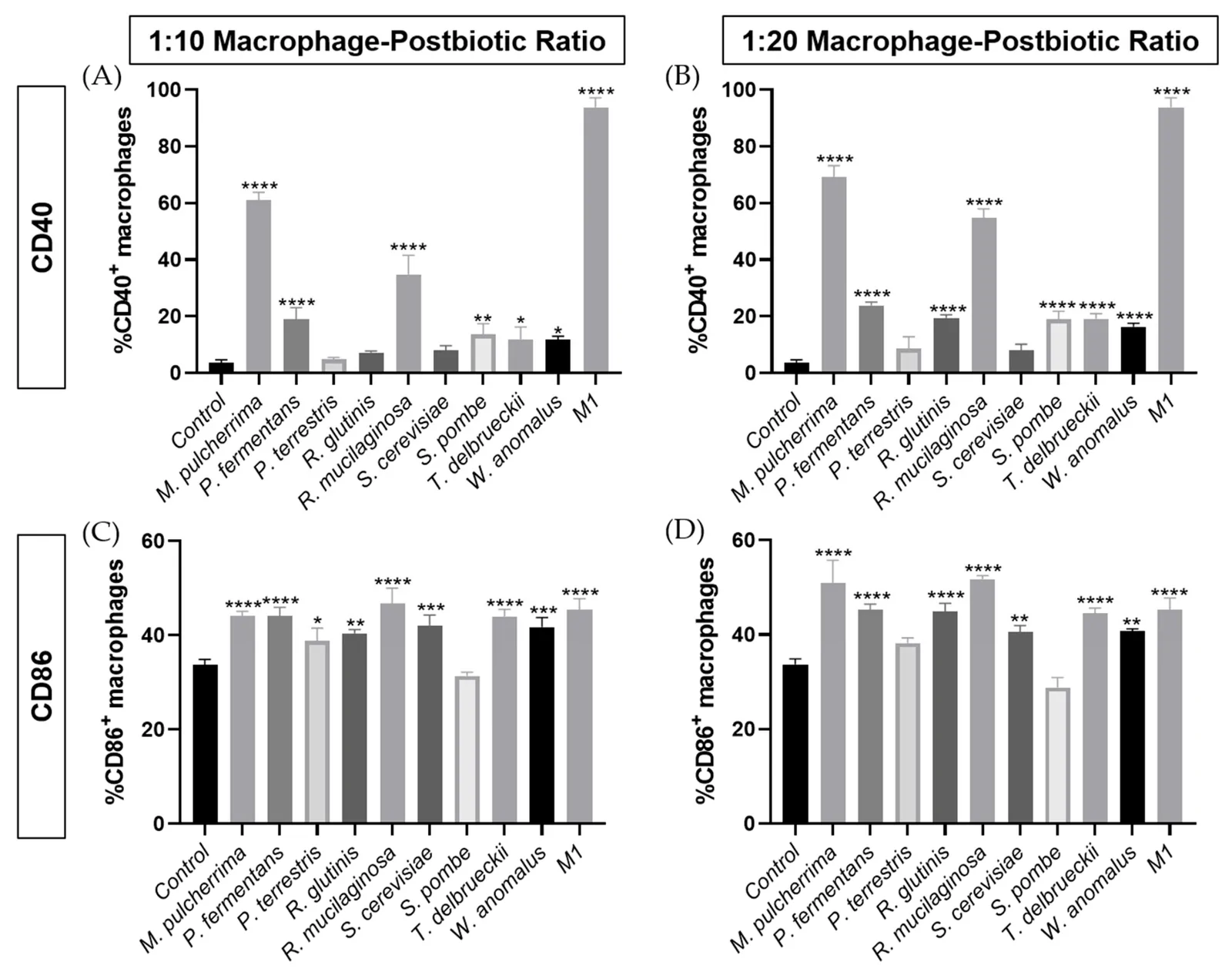
CD40 and CD86 surface expression in RAW 264.7 macrophages stimulated with heat-inactivated yeast postbiotics. CD40 surface expression following stimulation at (**A**) 1:10 and (**B**) 1:20 macrophage–postbiotic ratio. CD86 surface expression following stimulation at (**C**) 1:10 and (**D**) 1:20 macrophage–postbiotic ratio. Statistical increased significance was determined relative to the negative control (* *p* < 0.05, ** *p* < 0.01, *** *p* < 0.001, **** *p* < 0.0001). M1-polarized macrophages were included as positive control.

We sought to characterize the inflammatory response elicited by yeast postbiotics by analyzing two proinflammatory surface markers, CD40 and CD86, across two concentrations of heat-inactivated yeast postbiotics. As shown in Figure 4A,B, expression of CD40 was significantly increased following stimulation with *M. pulcherrima*, *P. fermentans*, *R. mucilaginosa*, *S. pombe*, *T. delbrueckii* and *W. anomalus*, being dose-dependent. At high concentrations (ratio 1:20), the number of species capable of inducing significant CD40 expression increased, with all tested species showing a response except *P. terrestris* and *S. cerevisiae*, which elicited little or no increase compared to unstimulated control. A similar concentration-dependent pattern was observed for CD86. A similar ratio-dependent pattern was observed for CD86. At the 1:10 ratio, all yeast postbiotics significantly increased CD86 expression relative to the control, with the exception of *S. pombe*. At the 1:20 ratio, CD86 expression also increased significantly for most species, except for *S. pombe* and *P. terrestris* (Figure 4C,D). This is consistent with prior evidence showing that extracellular vesicles from *S. cerevisiae*, cell wall components obtained from *C. albicans*, mannoproteins isolated from *Cryptococcus neoformans*, heat-inactivated *S. cerevisiae* cells, and β-glucans from *S. cerevisiae* induce increased expression of maturation markers such as CD40, CD86, CD80, and MHC-II in trained innate immune cells [20,21,22,23,84,85].

To further characterize the proinflammatory response suggested by the increased surface expression of CD40 and CD86, TNF-α production was assessed in macrophages stimulated with the same two concentrations of heat-inactivated yeast postbiotics. This cytokine was chosen as a functional readout given that its upregulation has been consistently reported following stimulation with *S. cerevisiae*-derived cell wall components and inactivated yeast cells, and its induction has been implicated in the establishment of trained innate immunity [49,86,87,88,89,90].

**Figure 5:**
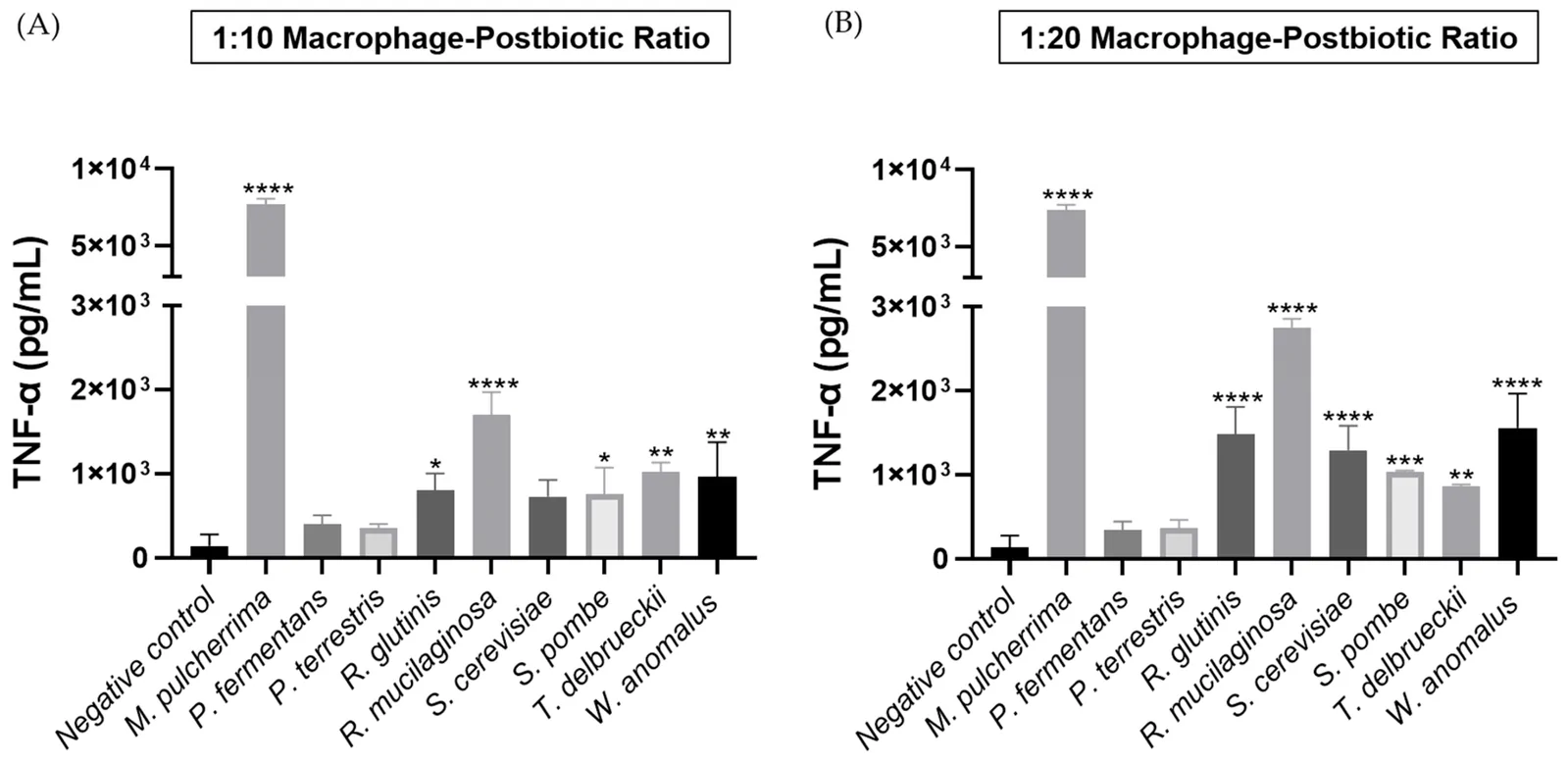
TNF-α production by RAW 264.7 macrophages stimulated with heat-inactivated yeast-derived postbiotics at two concentrations. TNF-α secretion was measured following stimulation with (**A**) 1:10 and (**B**) 1:20 macrophage–postbiotic ratio. Statistical significance was determined relative to the negative control (* *p* < 0.05, ** *p* < 0.01, *** *p* < 0.001, **** *p* < 0.0001). M1-polarized macrophages were included as positive control.

Figure 5A shows a significant TNF-α production in macrophages stimulated with low concentrations (ratio 1:10), *M. pulcherrima*, *R. glutinis*, *R. mucilaginosa*, *S. pombe*, *T. delbrueckii* and *W. anomalus*. At high concentrations (ratio 1:20), TNF-α secretion was markedly elevated across a broader range of species, with *M. pulcherrima*, *R. glutinis*, *R. mucilaginosa*, *S. cerevisiae*, *S. pombe*, *T. delbrueckii* and *W. anomalus* all inducing significant cytokine production. Collectively, the upregulation of CD40 and CD86 surface markers, together with the significant TNF-α production observed across multiple species, demonstrates that heat-inactivated yeast postbiotics are capable of eliciting a coordinated proinflammatory macrophage response, supporting their immunomodulatory potential as postbiotic candidates.

### 3.6. Reduction in ETEC Adhesion by Yeast Postbiotics

**Figure 6:**
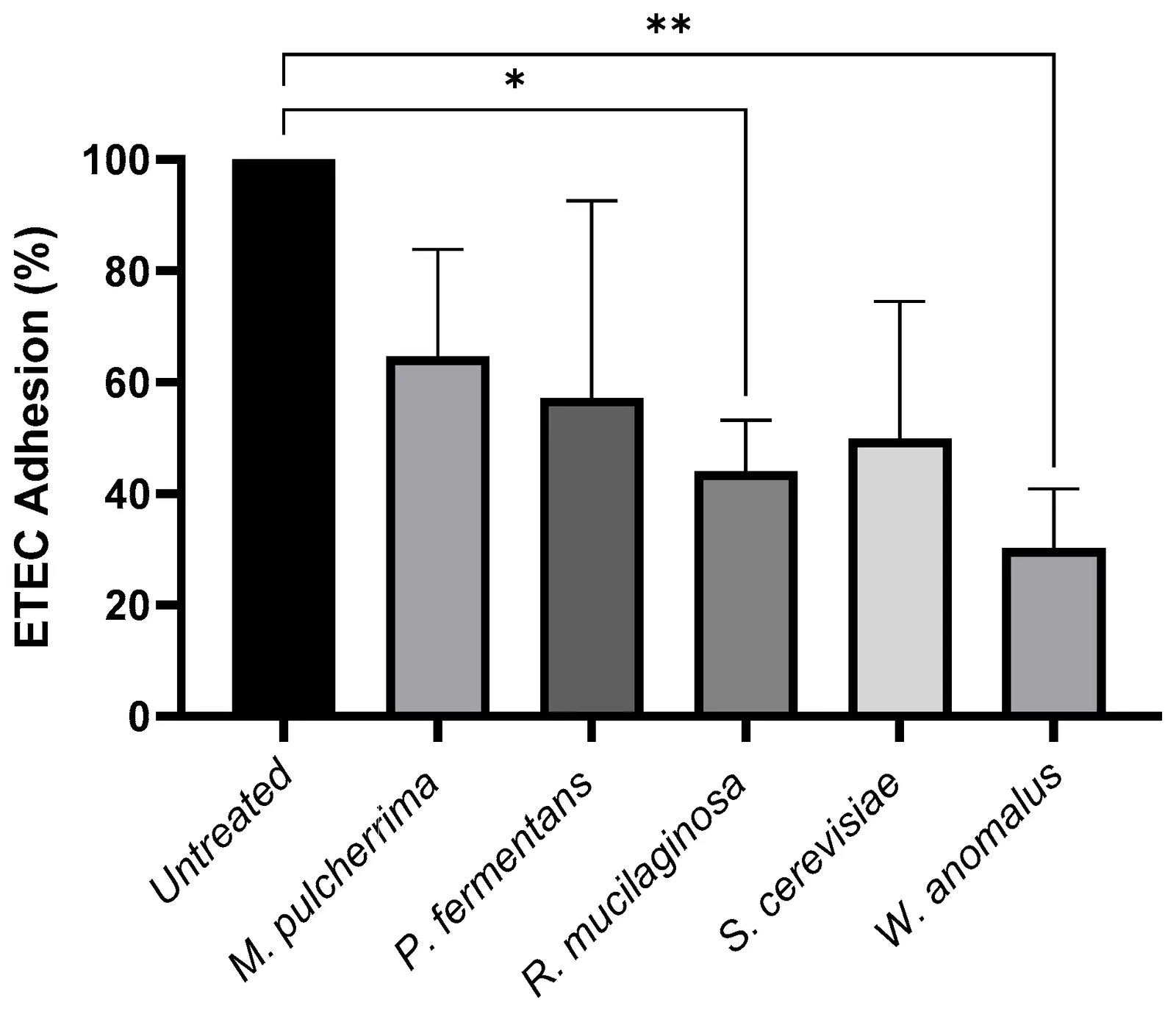
Effect of heat-inactivated yeast postbiotics on ETEC adhesion to Caco-2 cells. Percentage (%) of reduction in bacterial adhesion to Caco-2 cells in comparison to a yeast-free (Untreated) control is represented. (*, *p* < 0.05, ** *p* < 0.01). Error bars represent SEM (*n* = 3).

As previously mentioned, prior studies have reported that live yeast cells can directly interact with pathogens through the binding of yeast surface mannoproteins to bacterial type 1 fimbriae [18]. This interaction reduces bacterial adhesion to host cells, thereby preventing further infection within the host. Building on this, and based on previous results, we selected five heat-inactivated yeast postbiotics with demonstrated capacity to interact directly with enteric pathogens, such as enterotoxigenic *Escherichia coli* (ETEC) [18] and evaluated their ability to reduce ETEC adhesion to intestinal Caco-2 cells. Interestingly, all strains tested decreased ETEC adhesion to Caco-2 cells (approximately 36–70% of control); however, *W. anomalus* and *R. mucilaginosa* produced the most significant reductions, decreasing adhesion to approximately 56% and 70% of control, respectively (Figure 6). These results are consistent with prior evidence showing similar reductions in ETEC adhesion induced by yeast live cells from different species (*S. cerevisiae*, *P. fermentans* or *W. anomalus*) or dietary fibers obtained from *S. cerevisiae*, both in vitro and in a murine in vivo model [18,91,92]. Additionally, these investigations also reported reduced ETEC growth and toxin production following incubation with live *S. cerevisiae* or *S. cerevisiae* dietary fibers [91,92].

These findings are particularly relevant given their translational applicability for the prevention of gastrointestinal diseases in livestock. Type 1 fimbriae are highly conserved adhesins across *E. coli* strains regardless of host origin [93,94,95] and have been specifically shown to mediate adhesion of porcine F18ac^+^ ETEC strains [95], suggesting that this mechanism may also hold relevance for animal-associated ETEC. Indeed, ETEC infection is highly prevalent in weaned piglets, causing diarrhea, dehydration, growth retardation or even death [16]. Supporting this translational potential, several in vivo studies conducted on weaned piglets administered with different yeast postbiotics have shown decreased diarrhea index and survival rate [16]. Additionally, ETEC is also a common pathogen in neonatal ruminants, and research has demonstrated the beneficial effects of yeast-derived postbiotics on gut microbiota modulation, rendering the intestinal environment less hospitable to pathogens and reducing pathogen colonization [96]. Moreover, since type 1 fimbriae are not exclusive to ETEC, these postbiotics may represent a broader strategy against other type 1 fimbriae-bearing pathogens relevant to livestock health, as previously demonstrated for *Salmonella* using live yeast sharing the same mannoprotein-mediated binding mechanism [18].

In summary, this study provides evidence that heat-inactivated postbiotics derived from non-*Saccharomyces* yeast species are capable of eliciting a proinflammatory response and exerting antimicrobial activity against ETEC, supporting their potential as bioactive antimicrobial products for livestock applications. In this context, conducting further assays with livestock-derived cells, such as porcine or bovine intestinal cell lines (e.g., IPEC-J2 [97]) or macrophage/monocyte lines (e.g., BOMAC [98]) would provide valuable insights into host-specific cellular responses. Moreover, further in vivo assays are needed to validate their efficacy and safety ahead of commercial product development.

In addition, several other beneficial properties have been attributed to yeast-derived postbiotics, including enhancement of intestinal epithelial barrier integrity, promotion of beneficial microbiota species, and mycotoxin adsorption, further underscoring their multiple modes of action and their promising applicability [16]. More broadly, the use of yeast-derived postbiotics in livestock production remains an emerging field, underscoring the need to expand the range of yeast species tested, elucidate their specific mechanisms of action across different host species, and conduct long-term in vivo studies to determine optimal dosage, life stage administration and treatment duration [16].

## 4. Conclusions

Collectively, these findings contribute to the growing body of evidence supporting yeast-derived postbiotics as promising bioactive agents with immunomodulatory and antimicrobial properties, underscoring their preventive role against enteric infections and their potential application in livestock.

In particular, the present study highlights the immunomodulatory potential and antimicrobial activity of heat-inactivated yeast postbiotics derived from nine non-*Saccharomyces* species, reinforcing heat treatment as a suitable and scalable method for postbiotic production with potential real-world applications. Overall, results suggest that *R. mucilaginosa* and *W. anomalus* are the most promising postbiotic candidates for future studies. Nevertheless, further research is warranted to elucidate these mechanisms in vivo and determine optimal dosages for practical implementation.

## Figures and Tables

**Table 1:** Summary of the main beneficial properties reported in the literature for the nine yeast species selected in this study. These properties were identified as indicators of potential postbiotic activity and supported their selection for further evaluation.

Yeast Strains	Main Beneficial Properties	References
*Metschnikowia* *pulcherrima*	- Easily cultured in vitro - Adhesion, stress tolerance, aggregation, and antioxidant activity - Secretion of extracellular vesicles and mannoproteins - β-glucosidase and chitinase activity	[26,27,28,29,30,31,32]
*Pichia* *fermentans*	- Gastrointestinal tolerance, auto-aggregation, hydrophobicity, and antimicrobial activity - Cell wall mannoproteins and β-glucans - Secretion of extracellular vesicles - Immunomodulatory activity: β-glucans and extracellular vesicles are recognized by innate immune cells, enhancing the immune response	[29,30,33,34,35,36,37,38]
*Papiliotrema* *terrestris*	- Low-risk microorganism with favorable biosafety profile - Secretion of exopolysaccharides with antimicrobial activity	[39,40]
*Rhodotorula glutinis*	- Production of bioactive compounds: lipids, carotenoids, and exopolysaccharides - Enhanced innate immune response, growth promotion and reduced oxidative stress in Nile tilapia in vivo model	[41,42,43]
*Rhodotorula* *mucilaginosa*	- Gastrointestinal tolerance, auto-aggregation, hydrophobicity and antimicrobial activity - Enhanced innate immune response and protection observed in vivo in mice - Lipids, enzymes and carotenoid production - Probiotic and postbiotic properties in shrimp in vivo model: innate immunity activation, growth performance and body color	[44,45,46,47]
*Saccharomyces* *cerevisiae*	- Reference probiotic and postbiotic strain - Qualified Presumption of Safety (QPS) status - Secretion of extracellular vesicles - Immunomodulatory activity: yeast cell components or compounds promote cell activation, enhancing an innate or trained innate immune response	[17,29,48,49,50,51]
*Schizosaccharomyces pombe*	- Qualified Presumption of Safety (QPS) status - Gastrointestinal tolerance, auto-aggregation and antimicrobial activity	[48,52]
*Torulaspora* *delbrueckii*	- Gastrointestinal tolerance, cholesterol modulation, improve barrier function and stronger immune system - Secretion of extracellular vesicles	[29,36,53,54]
*Wickerhamomyces anomalus*	- Qualified presumption of safety (QPS) status - Gastrointestinal tolerance, antioxidant activity, and beneficial effects on gut health in animal models - Mannoproteins with antimicrobial effects - Production of exopolysaccharides	[36,48,55,56,57]

**Table 2:** Minimum conditions required for inactivation of stationary-phase yeast cultures by chemical (BEI/FA) and heat treatments.

	BEI/FA ^1^	Heat Treatment ^2^	
Yeast Strain	BEI Concentration (M), 24 h	Temperature (°C)	Time of Exposure (min)
*Metschnikowia pulcherrima*	0.2 ^3^	70	60
*Pichia fermentans*	0.2 ^3^	80	80
*Papiliotrema terrestris*	0.2 ^3^	80	80
*Rhodotorula glutinis*	0.2 ^3^	60 ^3^	60 ^3^
*Rhodotorula mucilaginosa*	0.2 ^3^	60 ^3^	60 ^3^
*Saccharomyces cerevisiae*	0.2 ^3^	60 ^3^	60 ^3^
*Schizosaccharomyces pombe*	0.1 ^3^	60 ^3^	60 ^3^
*Torulaspora delbrueckii*	0.1 ^3^	70	60
*Wickerhamomyces anomalus*	0.1 ^3^	60 ^3^	60 ^3^

^1^ Minimum effective BEI concentration (M) alongside a constant formaldehyde concentration applied over 24 h. ^2^ Minimum temperature (°C) and exposure time (min) necessary to achieve complete inactivation. ^3^ Minimum inactivation conditions reproduced from Cerdán-Alduán, 2025 [18].

## Data Availability

Supporting data cannot be made publicly available due to privacy restrictions. The data may be shared by the corresponding author upon reasonable request, subject to applicable privacy and confidentiality requirements.

## Appendix A

### Appendix A.3

**Table A1:** Determination of the presence of three major cell wall components. β-glucan content was determined by colorimetric assay and is expressed as percentage (%). Mannoprotein and chitin abundance was assessed by flow cytometry and are reported as mean fluorescence intensity (MFI).

Yeast Strains	β-Glucan (%)		Mannoprotein (MFI)		Chitin (MFI)	
	HT	BEI/FA	HT	BEI/FA	HT	BEI/FA
*M. pulcherrima*	1.52	0.95	341,994.19	281,909.21	31,670.49	37,395.78
*P. fermentans*	2.21	1.22	251,838.03	258,287.95	2078.77	3811.27
*P. terrestris*	2.41	1.17	46,369.51	51,782.09	14,824.38	33,250.87
*R. glutinis*	2.14	0.38	190,369.97	125,573.67	237,405.81	18,586.62
*R. mucilaginosa*	0.07	0.50	834,761.01	1,379,827.27	55,953.40	60,186.38
*S. cerevisiae*	1.78	1.42	585,286.27	325,754.56	26,524.85	30,499.11
*S. pombe*	0.40	0.80	23,761.87	31,362.51	442.89	2026.21
*T. delbrueckii*	1.34	1.61	80,347.84	43,794.46	56,128.18	28,024.18
*W. anomalus*	1.52	0.95	341,994.19	281,909.20	31,670.49	37,395.78
